# Association between dietary (poly)phenol intake and the ATHLOS Healthy Ageing Scale in the Polish arm of the HAPIEE study

**DOI:** 10.1007/s11357-024-01275-0

**Published:** 2024-07-10

**Authors:** Urszula Stepaniak, Giuseppe Grosso, Maciej Polak, Barbara Gradowicz-Prajsnar, Magdalena Kozela, Martin Bobak, Albert Sanchez-Niubo, Denes Stefler, Josep Maria Haro, Andrzej Pająk

**Affiliations:** 1 Department of Epidemiology and Population Studies, Jagiellonian University Medical College, Skawinska Street 8, 31-066 Krakow, Poland; 2 Department of Biomedical and Biotechnological Sciences, University of Catania, Catania, Italy; 3 Department of Epidemiology and Public Health, University College London, London, UK; 4 Centro de Investigación Biomédica en Red de Salud Mental, CIBERSAM, Madrid, Spain; 5 Department of Social Psychology and Quantitative Psychology, University of Barcelona, Barcelona, Spain; 6 Research, Innovation and Teaching Unit, Parc Sanitari Sant Joan de Déu, Sant Boi de Llobregat, Barcelona Spain

**Keywords:** (Poly)phenols, Healthy aging, Scale, Central and Eastern Europe

## Abstract

Inverse association between (poly)phenol intake and age-related disorders has been demonstrated; however, little is known whether they affect comprehensively assessed healthy aging. The aim of this study was to evaluate the associations between the intake of (poly)phenol (including selected classes and subclasses) and healthy aging scores related to biopsychosocial aspects of health and functioning. A cross-sectional study was performed using data on 9774 randomly selected citizens of Krakow (Poland) who were 45–69 years of age. Dietary (poly)phenol intake was evaluated using a food frequency questionnaire and matching food consumption data with the Phenol-Explorer database. The healthy aging scores were estimated from the ATHLOS Healthy Ageing Scale (HAS) developed by the Ageing Trajectories of Health-Longitudinal Opportunities and Synergies (ATHLOS) consortium. Beta coefficients were calculated using multivariable linear regression models. In multivariable adjusted models, there were significant positive associations between the ATHLOS HAS score and intake of total (poly)phenols (*b* per increase of 100 mg/day = 0.081; 95% CI, 0.050; 0.112) and among main classes of (poly)phenols with phenolic acids (*b* = 0.139; 95% CI, 0.098; 0.180). Intake of remaining classes of (poly)phenols (flavonoids, lignans, stilbenes, and others) was not related to the ATHLOS HAS score. Among individual classes studied, hydroxycinnamic acids, flavonols, flavones, and dihydrochalcones were associated with better healthy aging. The findings suggest the beneficial effect of total dietary (poly)phenol and some classes and subclasses of (poly)phenol intake in terms of healthy aging in Poland. These findings should be confirmed in other settings and with prospective data.

**Supplementary Information:**

The online version contains supplementary material available at 10.1007/s11357-024-01275-0.

## Introduction

In 2019, the proportion of people aged 65 years or more in 27 countries of the European Union was 20.3% of the total population, with growing trends estimated to reach nearly 30% in the next three decades [1]. From a public health perspective, the aging of the population is associated with important economic, health, and social challenges. In the World Health Organization (WHO) program, the Decade of Healthy Ageing (2020–2030), the need of research on healthy aging, particularly focused on the prevention of age-related diseases, has been emphasized [2]. Healthy aging is defined by the WHO as “a process of maintaining functional ability to enable wellbeing in older age” [2]. The rise in age-related non-communicable diseases, such as cardio-metabolic, neurodegenerative, and certain cancers (e.g., lung, breast, prostate), significantly affect the health status of the growing aging population, laying the ground for dramatic scenarios to be faced in the near future [3]. A major challenge for the health care systems will be to meet the health needs of the growing number of people with disabilities resulting from age-related disorders. Comparisons of healthy aging indicators have showed differences across European countries, with a substantial East–West gradient [4, 5]. While from a biological point of view the aging process is related to the immune function and, thus, partially influenced by genetic factors, the role of the environment and lifestyle seem to be responsible for the majority of differences between individuals [6–8]. Among lifestyle factors, diet has emerged as a modifiable lifestyle factor known to have an impact on aging [9, 10].

(Poly)phenols are a group of naturally occurring plant antioxidants that have been lately studied for their potential effects on human health [11, 12]. Based on their chemical structure, these compounds are categorized into phenolic acids, flavonoids, stilbenes, lignans, and other minor groups [11]. They are widely found in plants in which they provide protection against ultraviolet radiation or pathogen invasion [11]. (Poly)phenols have also demonstrated biological activities in human cells, including antioxidant, anti-inflammatory, anti-proliferative properties, pro-apoptotic activity, and hormonal regulation capacity, thus possibly playing a preventive role against age-related disorders and support healthy aging [13]. Other potential anti-aging mechanisms of (poly)phenols may include preventing cellular senescence, targeting microRNA, influencing nitric oxide (NO) bioavailability, and promoting mitochondrial function [13, 14]. (Poly)phenols have also been studied for their potential neuroprotective effects, such as improvement of cerebral blood flow and connectivity of the hippocampus, inducing neurogenesis, as well as reduction of oxidative stress and neuroinflammation related to the maintenance of cognitive functions [15, 16].

Studies on the association between dietary (poly)phenols and healthy aging indices in populations are emerging, but findings are inconclusive. In general, most results indicated a beneficial role of higher intake of (poly)phenols, like reducing mortality risk, preventing cognitive disorders, and delaying the biological aging process [17–23]; however, in some cohort studies, dietary (poly)phenols had no significant effect on mortality [24, 25], cognitive decline [26], or physical performance [27]. The ambiguity of the results obtained may depend on research methods used, different age and other characteristics of studied groups, diverse study designs, and methods of dietary assessment including various databases used for estimation of (poly)phenol content in the diet. Another reason may be the lack of a tool that adequately tackled the complex concept of healthy aging. Most previous research has examined only some of the characteristics of healthy aging, and no study assessing the relationship between dietary (poly)phenol intake and a universal indicator that could broadly capture a person’s healthy aging has been found.

The aim of this study was to assess the relationship of dietary (poly)phenol intake (including selected classes and subclasses) with healthy aging, assessed by the ATHLOS HAS, in the urban population of Poland.

## Methods

### Study sample

The HAPIEE (Health, Alcohol and Psychosocial factors in Eastern Europe) study is a prospective cohort study aiming to investigate psychosocial and dietary determinants of cardiovascular diseases (CVD) and other chronic conditions in Central and Eastern Europe. Details of the study design and methods have been published elsewhere [28]. For the present study, data from the Polish arm of the HAPIEE study, collected at baseline in 2002–2005 from individuals living in the municipality of Krakow (Poland), was used. A random population sample of 10,728 men and women aged 45–69 years, stratified by gender and 5-year age groups, was selected from the city population register. The response rate was 61% [28]. A standardized interview was first conducted at the participants’ home, and then they were invited to the clinic for a physical examination. The HAPIEE study was approved by ethics committees at the University College London and at the Jagiellonian University Medical College. All participants gave their written informed consent.

### Dietary assessment

Individual dietary habits were assessed using a semi-quantitative food frequency questionnaire (FFQ), based on the instrument developed by Willett et al., and subsequently modified for the Whitehall II study [29]. The FFQ consisted of 148 food and drink items, including coffee, tea, apples, and fruit juices. An instruction manual that included photographs to facilitate the estimation of portion sizes was used. Participants were asked how often, on average, they had consumed that amount of a particular food during the last 3 months, with nine responses ranging from “never or less than once per month” to “six or more times per day.” Moreover, participants were asked to include additional (other than listed in FFQ) foods and frequency of consumption by manual entry.

### Estimation of (poly)phenol intake

Data on the (poly)phenol content in foods were obtained from the Phenol-Explorer database (http://www.phenol-explorer.eu) [30]. The process of estimation of (poly)phenol intake has been described in detail elsewhere [31]. Briefly, food items of the FFQ were separated according to their ingredients and foods that contained no (poly)phenols were excluded from the analysis. The average food consumption was calculated (in g or ml) by following the standard portion sizes used in the study and then converted in 24-h intake. An advanced search was conducted in the Phenol-Explorer database to retrieve mean content values for all (poly)phenols contained in the foods obtained, and individual (poly)phenol intake from each food was calculated by multiplying the content of each (poly)phenol by the daily consumption of each food. Total (poly)phenol intake was calculated as the sum of all individual (poly)phenol intakes from all food sources encountered according to this process. In this study, we investigated exposure to total (poly)phenols and their main classes: phenolic acids, flavonoids, stilbenes, lignans, and others; the main subclasses of phenolic acids, including hydroxybenzoic acids and hydroxycinnamic acids; the main subclasses of flavonoids, including flavanols, flavonols, flavanones, flavones, anthocyanins, and dihydrochalcones.

### Measurement of healthy aging

Individual healthy aging was measured using the ATHLOS HAS, which is a novel tool developed by the ATHLOS consortium [32]. The score was constructed using the harmonized data from 16 cohorts from 38 different countries on six continents. Details on the construction of the ATHLOS HAS and its performance have been widely presented and discussed in earlier publications [5, 32]. Briefly, the scale was based on 41 biopsychosocial aspects of health and functioning covering domains on locomotion (walking, kneeling, lifting, climbing stairs, getting up from sitting down, stooping, etc.), cognition (orientation in time, memory, immediate and delayed recall, etc.), sensory (vision and hearing), vitality (energy, pain, etc.), and activities of daily living (getting in or out of bed, getting dressed, eating, preparing meals, shopping, bathing, using the toilet, housework, etc.) that imply interaction with the individual’s environment [5]. An item response theory (IRT) model was used to develop the scale, which indicated adequate goodness of fit as a unidimensional measure and high reliability. Scale values follow a normal distribution with a mean of 50 and a standard deviation of 10 points with higher values indicating better healthy aging. The validity of the scale against sociodemographics, health factors, and mortality has shown that the score well predicts the health status and could be useful in international aging studies [32, 33]. The HAPIEE study was one of the studies included in the harmonized ATHLOS mega dataset [32]. Complete data on the ATHLOS HAS was available for 9782 respondents. After the exclusion of participants with unreliable dietary intakes (i.e., < 500 kcal/day/ > 4500 kcal/day in women; < 800 kcal/day/ > 5000 kcal/day in men), a total sample of 9774 participants was included in the current analysis.

### Potential confounders

At baseline examination, a standard questionnaire was administered by trained nurses to collect data on age, gender, socioeconomic, and lifestyle characteristics. Education was categorized into two groups: (i) university and (ii) middle or lower. Marital status was categorized as (i) married/cohabited and (ii) single/widowed/divorced. Smoking was divided into three groups as (i) current smokers, (ii) ex-smokers, and (iii) non-smokers. According to leisure time physical activity, three groups were set: (i) 0 min/week, (ii) 1–149 min/week, and (iii) ≥ 150 min/week. Alcohol intake was assessed as a continuous variable (grams per year). History of CVD (i.e., self-reported history of myocardial infarction or stroke) was dichotomized. Based on standard measurements of body weight and height performed at the clinic, body mass index (BMI, kg/m^2^) was calculated. There were some missing data in the above variables, i.e., education (0.1%), marital status (0.2%), smoking (0.3%), alcohol intake (0.4%), history of CVD (0.8%), physical activity (5.2%), and BMI (12.0%).

### Statistical analysis

The quantitative variables were presented as mean and standard deviation (SD) or median and interquartile range (Q1–Q3). The Shapiro–Wilk test was used to test the assumption of normal distribution. Normally distributed variables were compared between groups using the *t*-test for an independent sample. Non-normally distributed variables were compared between groups using the Mann–Whitney *U* test. Categorical variables were described by percentages and compared using *χ*^2^ test. Total and individual classes and subclasses of (poly)phenol intake were adjusted for total energy intake (kcal/day) using the residual method [34].

Linear regression models were used to examine the associations between (poly)phenol intake and the ATHLOS HAS score. Results were presented as beta coefficient [according to average intakes, per increase of 100 mg/day of total (poly)phenols, flavonoids, phenolic acids, hydroxybenzoic acids, hydroxycinnamic acids, flavanols, flavonols, and flavanones; per 1 mg/day of stilbenes, flavones, anthocyanins, dihydrochalcones, and other minor (poly)phenols; per 10 µg/day of lignans] with 95% CI. Several sets of models were performed to take into account the influence of covariates on the investigated relationships. In the fully adjusted model, the following potential confounders were considered: age, sex, total energy intake, education, marital status, smoking, BMI, physical activity, history of cardiovascular disease (CVD). Additionally, standardized beta coefficients were reported.

Because of the two-stage nature of the baseline examination, the participation rate for the clinical examination was lower than for the interview. Thus, the number of participants included in the final multivariable model was lower (by approximately 12%), as the sample was restricted to participants without missing data on any of the covariates. Statistical analyses were performed using the Statistica version 13.0 software and IBM SPSS Statistics for Windows, version 27.0. Armonk, NY: IBM Corp. *P*-values < 0.05 were accepted as statistically significant.

## Results

Baseline characteristics for 9774 participants are presented in Table 1. The mean age of the total sample was 57.6 (SD 6.98) years; women accounted for 51.4%. The mean of the ATHLOS HAS score was 49.4 points (SD 9.13). Nearly 30% of respondents had a university education and most respondents (76.5%) were married or cohabited. Current smoking and lack of physical activity occurred in about 30% of the studied group. The mean BMI was 28.2 kg/m^2^. Positive history of CVD was rare (7.1%). The range of alcohol intake was from 0 to 1140 g per year. The mean intake of total (poly)phenol was 1651.7 mg/day. The main (poly)phenol groups were flavonoids (851.4 mg/day) and phenolic acids (702.0 mg/day). Consumption of stilbenes and lignans was very low (0.03 mg/day and 0.3 mg/day, respectively).

**Table 1:** Baseline characteristics of study participants

Variables	Total sample, *N* = 9774
Age [years], mean (SD)	57.6 (6.98)
Sex, *n* (%)	
Men	4752 (48.6)
Women	5022 (51.4)
The ATHLOS Healthy Ageing Scale score, mean (SD)	49.4 (9.13)
Education, *n* (%)	
Middle or lower	6980 (71.5)
University	2786 (28.5)
Marital status, *n* (%)	
Single, widowed, divorced	2290 (23.5)
Married, cohabited	7461 (76.5)
Smoking status, *n* (%)	
Current smoker	3138 (32.2)
Ex-smoker	2770 (28.4)
Non-smoker	3839 (39.4)
Physical activity groups, *n* (%)	
0 (min/week)	2770 (29.9)
1–149 (min/week)	1195 (12.9)
> 150 (min/week)	5304 (57.2)
History of cardiovascular disease, *n* (%)	
No	9006 (92.9)
Yes	687 (7.1)
BMI [kg/m^2^], mean (SD)	28.2 (4.61)
Energy intake [kcal], mean (SD)	2142.3 (639.96)
Alcohol consumption [g/year], (Me, Q1–Q3)	140 (0–1140)
Phenolic acids, mg/d (Me, Q1–Q3)	702.0 (446.8–1168.7)
Hydroxybenzoic acids, mg/d (Me, Q1–Q3)	90.1 (78.9–103.7)
Hydrossicynnamic acids, mg/d (Me, Q1–Q3)	613.9 (337.4–1070)
Flavonoids, mg/d (Me, Q1–Q3)	851.4 (677–1058.1)
Flavanols, mg/d (Me, Q1–Q3)	599.4 (461.8–774.3)
Flavonols, mg/d (Me, Q1–Q3)	100.8 (79.5–125.9)
Flavanones, mg/d (Me, Q1–Q3)	79.8 (44–131.3)
Flavones, mg/d (Me, Q1–Q3)	5.8 (3.3–9.8)
Anthocyanins, mg/d, (Me, Q1–Q3)	11.2 (7.2–19.3)
Dihydrochalcones, mg/d (Me, Q1–Q3)	9 (4.3–16.2)
Lignans, mg/d (Me, Q1–Q3)	0.3 (0.2–0.4)
Stilbenes, mg/d (Me, Q1–Q3)	0.03 (0.011–0.086)
Others, mg/d (Me, Q1–Q3)	23.8 (11.7–43.8)
Total (poly)phenols, mg/d (Me, Q1–Q3)	1651.7 (1326.8–2064.1)

*SD* standard deviation, *Me* median, *Q1* first quartile, *Q3* third quartile, *mg/d* milligram per day

Results of the linear regression model are presented in Table 2. In the age- and sex-adjusted models, a positive association between intake of total (poly)phenols, phenolic acids, flavonoids, stilbenes, and other (poly)phenols and the ATHLOS HAS scores was found. Intake of lignans was not related to the ATHLOS HAS scores. Among subclasses of phenolic acids, intake of hydrossicynnamic acids was positively associated with healthy aging. Among subclasses of flavonoids, intake of flavanols, flavonols, flavones, and dihydrochalcones was positively associated with healthy aging.

**Table 2:** Association between (poly)phenol intake and the ATHLOS Healthy Ageing Scale—results of the multivariable linear regressions

(Poly)phenols		Beta	95% CI	*P*	Standardized beta coefficient
Phenolic acids^a^	Model 1^d^	0.137	0.099; 0.175	< 0.001	0.069
	Model 2^e^	0.148	0.111; 0.185	< 0.001	0.075
	Model 3^f^	0.139	0.098; 0.180	< 0.001	0.070
Hydroxybenzoic acids^a^	Model 1^d^	0.157	− 0.230; 0.544	0.427	0.008
	Model 2^e^	0.246	− 0.139; 0.631	0.210	0.012
	Model 3^f^	− 0.063	− 0.467; 0.342	0.762	− 0.003
Hydrossicynnamic acids^a^	Model 1^d^	0.130	0.093; 0.167	< 0.001	0.066
	Model 2^e^	0.145	0.108; 0.182	< 0.001	0.074
	Model 3^f^	0.138	0.098; 0.178	< 0.001	0.071
Flavonoids^a^	Model 1^d^	0.080	0.031; 0.129	0.001	0.031
	Model 2^e^	0.077	0.028; 0.126	0.002	0.029
	Model 3^f^	0.002	− 0.050; 0.054	0.955	0.001
Flavanols^a^	Model 1^d^	0.074	0.017; 0.131	0.011	0.024
	Model 2^e^	0.072	0.015; 0.129	0.013	0.024
	Model 3^f^	− 0.012	− 0.073; 0.048	0.693	− 0.004
Flavonols^a^	Model 1^d^	1.249	0.834; 1.664	< 0.001	0.057
	Model 2^e^	1.257	0.845; 1.669	< 0.001	0.057
	Model 3^f^	0.830	0.401; 1.259	< 0.001	0.038
Flavanones^a^	Model 1^d^	0.042	− 0.166; 0.250	0.694	0.004
	Model 2^e^	0.080	− 0.127; 0.287	0.447	0.007
	Model 3^f^	0.081	− 0.138; 0.300	0.470	0.007
Flavones^b^	Model 1^d^	0.053	0.024; 0.082	< 0.001	0.035
	Model 2^e^	0.053	0.024; 0.081	< 0.001	0.035
	Model 3^f^	0.041	0.010; 0.071	0.009	0.026
Anthocyanins^b^	Model 1^d^	− 0.001	− 0.003; 0.002	0.609	− 0.005
	Model 2^e^	− 0.001	− 0.004; 0.001	0.313	− 0.010
	Model 3^f^	− 0.002	− 0.005; 0.000	0.076	− 0.018
Dihydrochalcones^b^	Model 1^d^	0.055	0.037; 0.074	< 0.001	0.057
	Model 2^e^	0.052	0.034; 0.071	< 0.001	0.054
	Model 3^f^	0.040	0.021; 0.060	< 0.001	0.042
Lignans^c^	Model 1^d^	− 0.006	− 0.174; 0.161	0.941	− 0.001
	Model 2^e^	− 0.003	− 0.170; 0.163	0.967	0.000
	Model 3^f^	− 0.093	− 0.272; 0.087	0.312	− 0.010
Stilbenes^b^	Model 1^d^	0.528	0.250; 0.806	< 0.001	0.036
	Model 2^e^	0.491	0.214; 0.767	0.001	0.033
	Model 3^f^	0.245	− 0.054; 0.543	0.108	0.016
Others^b^	Model 1^d^	0.008	0.002; 0.014	0.005	0.027
	Model 2^e^	0.007	0.001; 0.012	0.020	0.022
	Model 3^f^	− 0.003	− 0.009; 0.003	0.359	− 0.009
Total (poly)phenols^a^	Model 1^d^	0.115	0.086; 0.144	< 0.001	0.075
	Model 2^e^	0.118	0.089; 0.147	< 0.001	0.077
	Model 3^f^	0.081	0.050; 0.112	< 0.001	0.053

^a^Per 100 mg/day

^b^Per 1 mg/day

^c^Per 10 µg/day

^d^Model 1 adjusted for age and sex

^e^Model 2 adjusted for age, sex, and energy intake

^f^Model 3 adjusted for age, sex, energy intake, education, marital status, smoking, physical activity, BMI, and history of CVD

**Fig. 1:**
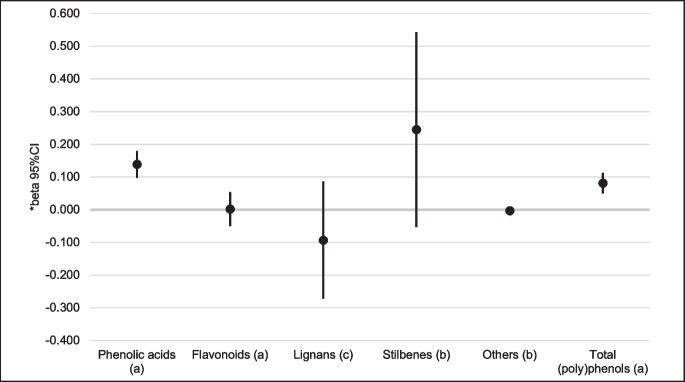
Association between main classes of (poly)phenols and total (poly)phenols and the ATHLOS Healthy Ageing Scale—results of the multivariable linear regressions. *Adjusted for age, sex, energy intake, education, marital status, smoking, physical activity, BMI, and history of CVD. (a) Per 100 mg/day; (b) per 1 mg/day; (c) per 10 µg/day

In the fully adjusted models, a significant positive association between intake of total (poly)phenols (*b* = 0.081; 95% CI, 0.050; 0.112) and the ATHLOS HAS scores was found. Among the main classes of (poly)phenols, a positive association between intake of phenolic acids (*b* = 0.139; 95% CI, 0.098; 0.180) and the ATHLOS HAS scores was found, while the intake of remaining classes of (poly)phenols (flavonoids, lignans, stilbenes, and others) was not related to the ATHLOS HAS scores (Table 2, Fig. 1).

Among individual subclasses of phenolic acids, there was a significant positive association between intake of hydrossicynnamic acids (*b* = 0.138; 95% CI, 0.098; 0.178) and the ATHLOS HAS scores. Among studied subclasses of flavonoids, relationship between the ATHLOS HAS scores and intake of flavonols (*b* = 0.830; 95% CI, 0.401; 1.259), flavones (*b* = 0.041; 95% CI, 0.010; 0.071), and dihydrochalcones (*b* = 0.040; 95% CI, 0.021; 0.060) was observed, with the strongest effect demonstrated with dihydrochalcones.

Additional adjustment for alcohol intake, intake of saturated fatty acids, and protein intake did not change the results (Online Resource 1).

## Discussion

The present study reports on a significant positive association between total dietary (poly)phenol intake and the ATHLOS HAS scores, which is mainly due to the significant effect of phenolic acids. Among individual classes studied, hydroxycinnamic acids among phenolic acids and flavonols, flavones, and dihydrochalcones among flavonoids were associated with better healthy aging.

No previous study has ever put in relation the dietary consumption of (poly)phenols and comprehensive measure of healthy aging. In general, findings from studies exploring the association between adherence to dietary patterns high in (poly)phenols, such as the Mediterranean diet, and healthy aging measured with composite scores are in line with a consistent positive relation [35]. Concerning studies specifically investigating the intake of (poly)phenols, higher consumption of total (poly)phenol was associated with lower mortality [19, 20]; moreover, comprehensive evidence of the literature supports the association between higher intake of flavonoids and reduced mortality, especially from CVD [21]. Also, there is rather homogeneity of results from observational studies on most common non-communicable diseases relating higher intake of (poly)phenols with lower risk of type 2 diabetes [36, 37], hypertension [38], CVD risk [37, 39], certain cancers [40], and cognitive decline [22]. In line with these results are also previous findings from the same HAPIEE cohort, in which higher intake of total (poly)phenols was associated with better health status, such as lower occurrence of metabolic syndrome [41], lower risk of type 2 diabetes [42], and hypertension (in women) [43]. The beneficial effect of dietary total (poly)phenol consumption on more specific age-related outcomes was also observed in other individual studies. In the cross-sectional analysis of 4592 participants aged 35 + in Italy, it was found that a diet rich in (poly)phenols was associated with decelerated biological aging, in particular, slower biological aging was inversely associated with the (poly)phenol antioxidant content (PAC) score of the diet [17]. Nonetheless, in the Invecchiare in Chianti study conducted on 811 participants aged 65 years and older, higher total dietary (poly)phenol intake was not significantly associated with prevalence of frailty and prefrailty [44], risk of cognitive decline [26], risk of substantial decline in physical performance in 9-year follow-up observation [27], or with all-cause mortality in 12-year observation [24].

Among the main classes of (poly)phenol, intake of phenolic acids (specifically hydroxycinnamic acids) was positively associated with the ATHLOS HAS scores. Studies conducted specifically on phenolic acids are rather scarce. However, the few existing reports suggest that a higher intake of phenolic acids may be associated with better cardio-metabolic fit and cognitive status among the older population [45–47]. These results are also in line with previous findings of the Polish arm of the HAPIEE cohort which showed that phenolic acids and, specifically, hydroxycinnamic acids were associated with a better health profile, including lower odds of metabolic syndrome [41], lower incidence of hypertension in women [43], and lower risk of type 2 diabetes [42]. In other studies, phenolic acids and particularly hydroxycinnamic acids were associated with lower mortality in Spain [18] and lower prevalence of insulin resistance in adults at age 40–70 years in Israel [48]. Also, in a study of Italian adults above 50 years old, those in the highest quartile of total phenolic acid and hydroxycinnamic acids intake were less likely to have impaired cognitive status [45]. The main food source of hydroxycinnamic acids in the present cohort has been estimated to be coffee [31]: hence, this observation may also provide the rationale for the benefits of moderate coffee consumption and non-communicable diseases reported in the scientific literature [49]. Broadly, coffee drinking has been found to be associated with better physical functioning outcomes in adults above 40 years [50]. Concerning the Polish arm of the HAPIEE cohort, previous findings showed that coffee intake was related to better health outcomes like metabolic syndrome and most of its components [51]; moreover, consumption of 3–4 cups of coffee per day was associated with lower mortality risk [52].

Results of other research regarding the relation between total flavonoids and age-related health outcomes are less consistent. In some studies, total flavonoid intake was not associated with all-cause mortality [25, 53], while in others, the beneficial effect of total flavonoid intake on decrease mortality risk [21, 54], non-fatal cardiovascular events [25], cognitive status [55], or the biological aging process [23, 56] was found. The mean intake of total flavonoids was relatively high in our cohort compared with other studies, but the main food sources were different. In our study, the major contributors to flavonoid intake were tea, chocolate, and apples [31], whereas in the Italian population, fruits, red wine, and vegetables [25, 57]; in the US NHANES study, it was tea, citrus fruit and citrus fruit juices, berries, wine, and vegetables [58]. The lack of significant effect of total flavonoids in our study may result from low consumption of vegetables and fruits in the Polish population [29]. However, regarding the individual subclasses of flavonoids, some of them were associated with the ATHLOS HAS scores. In the present study, a positive association between flavonol intake and the ATHLOS HAS scores was observed, which is in line with results of other studies [18, 21, 53, 59]. The main food source of flavonols in our sample was tea and vegetables, such as onions and spinach [31]. Additionally, consumption of flavones was positively associated with the ATHLOS HAS scores, which is consistent with results of some other studies evaluating the relationship between flavones and health benefits [23, 42, 56, 60]. Moreover, a positive association between dihydrochalcones and the ATHLOS HAS scores was reported. As the main food source of dihydrochalcones in our cohort were apples (93%) and apple juice (7%) [31], thus significant association may result from the relatively high consumption of apples in Poland, which are the most commonly consumed fruits [61]. The beneficial effect of dihydrochalcones on health status has also been demonstrated in other studies [59, 60].

From a mechanistic point of view, several hypotheses have been developed to explain the potential benefits of including (poly)phenols in the diet in order to reduce the risk of age-related non-communicable diseases [62]. (Poly)phenols exert antioxidant properties in plants, while their mechanisms of action in humans seem to be more complicated. Indeed, (poly)phenols exert their antioxidant activity through the inhibition of enzymes involved in ROS production (i.e., xanthine oxidase and NADPH oxidase (NOX)) and the upregulation of genes coding for antioxidant proteins, like superoxide dismutase (SOD), catalase, and glutathione peroxidase (Gpx) [63]. (Poly)phenols are also implicated in the regulation of different pathways such as phosphatidylinositide 3-kinases/protein kinase B (PI3K/AkT), inhibitor of kappa kinase/c-Jun amino-terminal kinases (IKK/JNK), mammalian target of rapamycin complex 1 (mTORC1), and JAK/STAT [64]. Moreover, (poly)phenols can suppress pro-inflammatory gene expression, through the inhibition of Toll-like receptor activity [63], as well as inactivating nuclear factor kappa (NF-κB) and the modulation of mitogen-activated protein kinase (MAPK) and arachidonic acid pathways [64]. However, most studies reported such molecular effects on native compounds, while extensive evidence suggests that (poly)phenols are highly transformed in the colon by the gut microbiota, leading to the absorption of metabolites exerting different effects on various molecular targets [65]. Interestingly, high content of (poly)phenols in the diet is able to exert positive modification on the gut microbiota itself through a synergistic interaction [66], promoting bacterial diversity and variety, increasing families associated with lower risk of chronic diseases (such as *Lactobacillus* and *Bifidobacterium*), and decreasing those observed to be potentially pro-inflammatory (including *Bacteroides*, *Clostridium*, and *Staphylococcus* genera) [67]. Such observed effects may play a role in systemic inflammation and also provide benefits in the central nervous system through communication via the gut-brain axis and potential inhibition of neuroinflammation, promotion of neural plasticity, mitochondrial health, and ultimately lower risk of neurodegenerative conditions. Although no specific mechanism has been unequivocally identified to explain the findings retrieved in the observational study, it is likely that a synergistic action between direct and indirect effects may play a role in the overall health status of older adults and the prevention of age-related chronic non-communicable diseases.

The present study has several strengths. It was conducted on a large random sample with a reliable assessment of individual diet. Next, we used the ATHLOS HAS, which allowed us to assess respondents’ healthy aging more comprehensively than through partial age-related health outcomes and which was shown as a useful tool for the assessment of the future health trajectories of the older population [33]. Further, in the exposure assessment, we included different subtypes of (poly)phenols rather than just total (poly)phenols. Finally, we included a wide range of covariates to adjust the studied relationship.

The findings of this study should be considered in light of some limitations. First, because of the cross-sectional design, we cannot exclude the possibility of reverse causation and that certain age-related conditions may influence participants’ dietary habits. To clarify this ambiguity, further research with a prospective study design is recommended. Second, some (poly)phenol-rich foods, such as herbs and spices, may not have been entirely captured by the FFQ, and the (poly)phenol intake could have been underestimated in our study.

Next, we do not have data on the bioavailability of the (poly)phenols. The beneficial action of (poly)phenols on human health depends not only on their content in foods but also on their stability, microbiota, and digestive enzymes and is influenced by various factors such as interactions with other compounds present in foods. Recent research suggested that interactions of (poly)phenols with proteins, lipids, carbohydrates, or other compounds present in foods may affect (poly)phenol bioaccessibility and bioavailability. For example, protein-rich meals are likely to cause detrimental effects on (poly)phenol bioaccessibility [68, 69]. The diet of our sample is characterized by high protein intake [29] which could have weakened the beneficial effect of (poly)phenols. However, adjustment for protein intake did not change the results. Finally, we aimed to adjust for the most important confounders in the analysis, but still, some residual confounding is possible. Due to a lack of data on medications or nutritional supplements taken by the enrolled subjects, we could not include it into the analysis. Additionally, as the study consisted of an urban population only, the results may not be generalizable to other, rural populations, or the Polish population as a whole.

In conclusion, the results of this large cross-sectional study indicate the beneficial effect of total dietary (poly)phenol and some classes and subclasses of (poly)phenol intake in terms of healthy aging in the CEE population.

## Supplementary Information

Below is the link to the electronic supplementary material.

Supplementary file1 (DOCX 29.9 KB)
