# Supplementary material for: Association between dietary (poly)phenol intake and the ATHLOS Healthy Ageing Scale in the Polish arm of the HAPIEE study
Source: GeroScience. 2024 Jul 10;47(3):3241–53. doi: 10.1007/s11357-024-01275-0 (PMC12181526; doi:10.1007/s11357-024-01275-0)
Supplement: Supplementary file 1 — Supplementary file1 (DOCX 29.9 KB) [file 11357_2024_1275_MOESM1_ESM.docx]

Association between dietary polyphenol intake and the ATHLOS Healthy Ageing Scale in the Polish arm of the HAPIEE study

GeroScience

Urszula Stepaniak, Giuseppe Grosso, Maciej Polak, Barbara Gradowicz-Prajsnar, Magdalena Kozela, Martin Bobak, Albert Sanchez-Niubo, Denes Stefler, Josep Maria Haro, Andrzej Pająk

The corresponding author:

Urszula Stepaniak

Department of Epidemiology and Population Studies,

Jagiellonian University Medical College,

Skawinska Street 8, 31-066 Krakow, Poland

phone number: 48 12 4332801;

e-mail: urszula.stepaniak@uj.edu.pl

Online Resource 1. Association between (poly)phenols intake and the ATHLOS Healthy Ageing Scale – results of the multivariable linear regressions.

| (Poly)phenols | beta | 95% CI | p | Standardized beta coefficient | 95% CI |
| --- | --- | --- | --- | --- | --- |
| Phenolic acids^a^ |  |  |  |  |  |
| Model 1 | 0.196 | 0.157; 0.235 | <0.001 | 0.100 | 0.080; 0.119 |
| Model 2 | 0.137 | 0.099; 0.175 | <0.001 | 0.069 | 0.050; 0.089 |
| Model 3 | 0.148 | 0.111; 0.185 | <0.001 | 0.075 | 0.056; 0.094 |
| Model 4 | 0.139 | 0.098; 0.180 | <0.001 | 0.070 | 0.050; 0.091 |
| Model 5 | 0.139 | 0.098; 0.180 | <0.001 | 0.071 | 0.050; 0.091 |
| Model 6 | 0.138 | 0.097; 0.179 | <0.001 | 0.070 | 0.050; 0.091 |
| Model 7 | 0,141 | 0,100; 0,182 | <0,001 | 0,072 | 0,051; 0,092 |
| Hydroxibenzoic acids^a^ |  |  |  |  |  |
| Model 1 | 0.205 | -0.202; 0.612 | 0.324 | 0.010 | -0.010; 0.030 |
| Model 2 | 0.157 | -0.230; 0.544 | 0.427 | 0.008 | -0.011; 0.026 |
| Model 3 | 0.246 | -0.139; 0.631 | 0.210 | 0.012 | -0.007; 0.031 |
| Model 4 | -0.063 | -0.467; 0.342 | 0.762 | -0.003 | -0.023; 0.017 |
| Model 5 | -0.060 | -0.465; 0.345 | 0.770 | -0.003 | -0.023; 0.017 |
| Model 6 | -0.058 | -0.463; 0.347 | 0.781 | -0.003 | -0.023; 0.017 |
| Model 7 | -0,056 | -0,460; 0,349 | 0,788 | -0,003 | -0,023; 0,017 |
| Hydrossicynnamic acids^a^ |  |  |  |  |  |
| Model 1 | 0.186 | 0.147; 0.225 | <0.001 | 0.095 | 0.075; 0.115 |
| Model 2 | 0.130 | 0.093; 0.167 | <0.001 | 0.066 | 0.047; 0.086 |
| Model 3 | 0.145 | 0.108; 0.182 | <0.001 | 0.074 | 0.055; 0.093 |
| Model 4 | 0.138 | 0.098; 0.178 | <0.001 | 0.071 | 0.050; 0.091 |
| Model 5 | 0.139 | 0.099; 0.179 | <0.001 | 0.071 | 0.050; 0.092 |
| Model 6 | 0.138 | 0.097; 0.179 | <0.001 | 0.071 | 0.050; 0.091 |
| Model 7 | 0,141 | 0,101; 0,181 | <0,001 | 0,072 | 0,051; 0,093 |
| Flavonoids^a^ |  |  |  |  |  |
| Model 1 | 0.067 | 0.015; 0.119 | 0.011 | 0.026 | 0.006; 0.046 |
| Model 2 | 0.080 | 0.031; 0.129 | 0.001 | 0.031 | 0.012; 0.050 |
| Model 3 | 0.077 | 0.028; 0.126 | 0.002 | 0.029 | 0.011; 0.048 |
| Model 4 | 0.002 | -0.050; 0.054 | 0.955 | 0.001 | -0.019; 0.020 |
| Model 5 | -0.001 | -0.053; 0.051 | 0.977 | 0.000 | -0.020; 0.020 |
| Model 6 | -0.001 | -0.053; 0.051 | 0.973 | 0.000 | -0.020; 0.020 |
| Model 7 | 0,012 | -0,041; 0,065 | 0,650 | 0,005 | -0,015; 0,025 |
| Flavanols^a^ |  |  |  |  |  |
| Model 1 | 0.081 | 0.021; 0.141 | 0.008 | 0.027 | 0.007; 0.047 |
| Model 2 | 0.074 | 0.017; 0.131 | 0.011 | 0.024 | 0.006; 0.043 |
| Model 3 | 0.072 | 0.015; 0.129 | 0.013 | 0.024 | 0.005; 0.043 |
| Model 4 | -0.012 | -0.073; 0.048 | 0.693 | -0.004 | -0.024; 0.016 |
| Model 5 | -0.016 | -0.076; 0.045 | 0.614 | -0.005 | -0.025; 0.015 |
| Model 6 | -0.019 | -0.080; 0.042 | 0.545 | -0.006 | -0.026; 0.014 |
| Model 7 | -0,002 | -0,064; 0,059 | 0,945 | -0,001 | -0,021; 0,020 |
| Flavonols^a^ |  |  |  |  |  |
| Model 1 | 0.889 | 0.453; 1.325 | <0.001 | 0.040 | 0.021; 0.060 |
| Model 2 | 1.249 | 0.834; 1.664 | <0.001 | 0.057 | 0.038; 0.076 |
| Model 3 | 1.257 | 0.845; 1.669 | <0.001 | 0.057 | 0.038; 0.076 |
| Model 4 | 0.830 | 0.401; 1.259 | <0.001 | 0.038 | 0.019; 0.058 |
| Model 5 | 0.828 | 0.398; 1.258 | <0.001 | 0.038 | 0.018; 0.058 |
| Model 6 | 0.871 | 0.437; 1.305 | <0.001 | 0.040 | 0.020; 0.060 |
| Model 7 | 0,831 | 0,397; 1,265 | <0,001 | 0,038 | 0,018; 0,059 |
| Flavanones^a^ |  |  |  |  |  |
| Model 1 | -0.019 | -0.238; 0.200 | 0.863 | -0.002 | -0.022; 0.018 |
| Model 2 | 0.042 | -0.166; 0.250 | 0.694 | 0.004 | -0.015; 0.023 |
| Model 3 | 0.080 | -0.127; 0.287 | 0.447 | 0.007 | -0.011; 0.026 |
| Model 4 | 0.081 | -0.138; 0.300 | 0.470 | 0.007 | -0.012; 0.027 |
| Model 5 | 0.086 | -0.134; 0.306 | 0.441 | 0.008 | -0.012; 0.028 |
| Model 6 | 0.106 | -0.117; 0.329 | 0.351 | 0.010 | -0.011; 0.030 |
| Model 7 | 0,115 | -0,107; 0,337 | 0,310 | 0,010 | -0,010; 0,030 |
| Flavones^b^ |  |  |  |  |  |
| Model 1 | 0.021 | -0.009; 0.051 | 0.171 | 0.014 | -0.006; 0.034 |
| Model 2 | 0.053 | 0.024; 0.082 | <0.001 | 0.035 | 0.016; 0.054 |
| Model 3 | 0.053 | 0.024; 0.081 | <0.001 | 0.035 | 0.016; 0.053 |
| Model 4 | 0.041 | 0.010; 0.071 | 0.009 | 0.026 | 0.007; 0.046 |
| Model 5 | 0.041 | 0.010; 0.072 | 0.009 | 0.027 | 0.007; 0.047 |
| Model 6 | 0.046 | 0.015; 0.078 | 0.004 | 0.030 | 0.010; 0.051 |
| Model 7 | 0,046 | 0,015; 0,078 | 0,004 | 0,030 | 0,010; 0,050 |
| Anthocyanins^b^ |  |  |  |  |  |
| Model 1 | -0.002 | -0.004; 0.001 | 0.143 | -0.015 | -0.035; 0.005 |
| Model 2 | -0.001 | -0.003; 0.002 | 0.609 | -0.005 | -0.024; 0.014 |
| Model 3 | -0.001 | -0.004; 0.001 | 0.313 | -0.010 | -0.028; 0.009 |
| Model 4 | -0.002 | -0.005; 0.000 | 0.076 | -0.018 | -0.038; 0.002 |
| Model 5 | -0.002 | -0.005; 0.000 | 0.074 | -0.018 | -0.038; 0.002 |
| Model 6 | -0.002 | -0.005; 0.000 | 0.086 | -0.017 | -0.037; 0.002 |
| Model 7 | -0,002 | -0,005; 0,000 | 0,109 | -0,016 | -0,036; 0,004 |
| Dihydrochalcones^b^ |  |  |  |  |  |
| Model 1 | 0.004 | -0.016; 0.023 | 0.715 | 0.004 | -0.016; 0.024 |
| Model 2 | 0.055 | 0.037; 0.074 | <0.001 | 0.057 | 0.038; 0.076 |
| Model 3 | 0.052 | 0.034; 0.071 | <0.001 | 0.054 | 0.035; 0.073 |
| Model 4 | 0.040 | 0.021; 0.060 | <0.001 | 0.042 | 0.022; 0.062 |
| Model 5 | 0.041 | 0.021; 0.060 | <0.001 | 0.042 | 0.022; 0.063 |
| Model 6 | 0.046 | 0.026; 0.066 | <0.001 | 0.048 | 0.027; 0.068 |
| Model 7 | 0,047 | 0,027; 0,067 | <0,001 | 0,049 | 0,028; 0,070 |
| Lignans^c^ |  |  |  |  |  |
| Model 1 | -0.026 | -0.202; 0.151 | 0.776 | -0.003 | -0.023; 0.017 |
| Model 2 | -0.006 | -0.174; 0.161 | 0.941 | -0.001 | -0.020; 0.018 |
| Model 3 | -0.003 | -0.170; 0.163 | 0.967 | 0.000 | -0.019; 0.018 |
| Model 4 | -0.093 | -0.272; 0.087 | 0.312 | -0.010 | -0.030; 0.010 |
| Model 5 | -0.093 | -0.272; 0.087 | 0.313 | -0.010 | -0.030; 0.010 |
| Model 6 | -0.094 | -0.274; 0.085 | 0.303 | -0.010 | -0.030; 0.009 |
| Model 7 | -0,094 | -0,273; 0,086 | 0,307 | -0,010 | -0,030; 0,009 |
| Stilbenes^b^ |  |  |  |  |  |
| Model 1 | 0.656 | 0.364; 0.949 | <0.001 | 0.044 | 0.025; 0.064 |
| Model 2 | 0.528 | 0.250; 0.806 | <0.001 | 0.036 | 0.017; 0.055 |
| Model 3 | 0.491 | 0.214; 0.767 | 0.001 | 0.033 | 0.015; 0.052 |
| Model 4 | 0.245 | -0.054; 0.543 | 0.108 | 0.016 | -0.004; 0.036 |
| Model 5 | 0.240 | -0.061; 0.540 | 0.118 | 0.016 | -0.004; 0.036 |
| Model 6 | 0.248 | -0.053; 0.549 | 0.106 | 0.016 | -0.004; 0.036 |
| Model 7 | 0,269 | -0,032; 0,570 | 0,080 | 0,018 | -0,002; 0,038 |
| Others^b^ |  |  |  |  |  |
| Model 1 | -0.006 | -0.012; 0.000 | 0.057 | -0.019 | -0.039; 0.001 |
| Model 2 | 0.008 | 0.002; 0.014 | 0.005 | 0.027 | 0.008; 0.046 |
| Model 3 | 0.007 | 0.001; 0.012 | 0.020 | 0.022 | 0.004; 0.041 |
| Model 4 | -0.003 | -0.009; 0.003 | 0.359 | -0.009 | -0.030; 0.011 |
| Model 5 | -0.003 | -0.009; 0.003 | 0.345 | -0.010 | -0.030; 0.010 |
| Model 6 | -0.002 | -0.008; 0.004 | 0.441 | -0.008 | -0.029; 0.013 |
| Model 7 | -0,003 | -0,009; 0,003 | 0,313 | -0,011 | -0,031; 0,010 |
| Total (poly)phenols^a^ |  |  |  |  |  |
| Model 1 | 0.143 | 0.113; 0.173 | <0.001 | 0.093 | 0.074; 0.113 |
| Model 2 | 0.115 | 0.086; 0.144 | <0.001 | 0.075 | 0.056; 0.094 |
| Model 3 | 0.118 | 0.089; 0.147 | <0.001 | 0.077 | 0.058; 0.096 |
| Model 4 | 0.081 | 0.050; 0.112 | <0.001 | 0.053 | 0.032; 0.073 |
| Model 5 | 0.080 | 0.049; 0.111 | <0.001 | 0.052 | 0.032; 0.073 |
| Model 6 | 0.080 | 0.049; 0.111 | <0.001 | 0.052 | 0.032; 0.072 |
| Model 7 | 0,087 | 0,056; 0,118 | <0,001 | 0,057 | 0,036; 0,077 |

^a^: per 100mg/day; ^b^: per 1mg/day; ^c^: per 10 µg/day

Model 1 crude

Model 2 adjusted for: age, sex

Model 3 adjusted for: age, sex, energy intake

Model 4 adjusted for: age, sex, energy intake, education, marital status, smoking, physical activity, BMI, history of CVD

Model 5 adjusted for: age, sex, energy intake, education, marital status, smoking, physical activity, BMI, history of CVD, alcohol intake

Model 6 adjusted for: age, sex, energy intake, education, marital status, smoking, physical activity, BMI, history of CVD, alcohol intake, saturated fatty acids intake

Model 7 adjusted for: age, sex, energy intake, education, marital status, smoking, physical activity, BMI, history of CVD, alcohol intake, saturated fatty acids intake, protein intake
